# Accuracy in Referrals to Gynecologic Oncologists Based on Clinical Presentation for Ovarian Mass

**DOI:** 10.3390/diagnostics10020106

**Published:** 2020-02-16

**Authors:** Katherine Jane C. Chua, Ricky D. Patel, Radhika Trivedi, Patricia Greenberg, Kyle Beiter, Thomas Magliaro, Ushma Patel, Joyce Varughese

**Affiliations:** 1 Department of Obstetrics and Gynecology, Saint Peter’s University Hospital, New Brunswick, NJ 08901, USA; ricky.patel@saintpetersuh.com (R.D.P.); upatel@saintpetersuh.com (U.P.); 2 Biostatistics and Epidemiology Services Center, Rutgers School of Public Health, Rutgers University, Piscataway, NJ 08854, USA; rt556@sph.rutgers.edu (R.T.); pjg134@sph.rutgers.edu (P.G.); 3 Department of Gynecology, Saint Peter’s Physician Associates, Somerset, NJ 08873, USA; kbeiter@saintpetersuh.com (K.B.); t.magliaromd@comcast.net (T.M.); 4 Capital Health Surgical Group, Gynecology Oncology, Pennington, NJ 08534, USA; Joycevarughese.MD@gmail.com

**Keywords:** ovarian mass, referral gynecologic oncologists

## Abstract

Ovarian cancer is one of the most lethal gynecological cancers in women due to late diagnosis. Despite technological advancements, experienced physicians have high sensitivities and specificities in subjective assessments when combining ultrasound findings and clinical history in analyzing adnexal masses. This study aims to demonstrate general obstetricians and gynecologists’ (OB/GYN) appropriateness in gynecologic oncologist referrals for malignant ovarian masses based on history and physical (H&P), imaging, and available tumor markers. Three board certified OB/GYNs were given 148 cases and determined whether or not they would refer them to a gynecologic oncologist. Results showed that OB/GYNs were 81–85% accurate in diagnosing patients with a benign or malignant disease. Among the malignant cases, reviewers had a high sensitivity ranging from 74–81% in appropriately referring a malignancy. In our study, OB/GYNs referred between 23–32% of ovarian masses to a gynecologic oncologist with only 9.5% of cases found to be malignant. Despite the high referral rates, generalists showed a high degree of sensitivity in accurately referring malignant diseases based solely on clinical experience and imaging studies, which could improve survival rates with early intervention by gynecologic oncologists.

## 1. Introduction

Ovarian cancer is one of the most lethal gynecological cancers in women due to the lack of symptoms in the early stages and limited screening tests applicable to the general population, leading to diagnosis at later stages. Although benign ovarian masses are more common than ovarian cancer, distinguishing benign ovarian masses from borderline and malignant masses is critical in increasing survival rates. Transvaginal ultrasound is the recommended diagnostic tool despite variation in sonographer skill, and serum tumor markers, although cost-effective, have limited sensitivity and specificity [1,2,3]. More recently, pre-surgical algorithms have become available to assess the probability of malignancy in patients, including the Risk of Ovarian Malignancy Algorithm (ROMA) and OVA-1 algorithm. CA 125 is the most commonly used tumor marker for ovarian cancer available; however, its use as a screening tool with simple cutoffs is unreliable [4,5]. An elevated CA 125 is caused by factors including endometriosis, race, smoking, caffeine intake, and a personal history of any cancer. On the other hand, a normal CA 125 cannot rule out an ovarian malignancy [5]. Referral to a gynecologic oncologist according to the ACOG/SGO Joint Opinion Guidelines remains appropriate for women with a pelvic mass, leading to the suspicion of an ovarian malignancy, and who have at least one of the following indicators: an elevated CA 125 (>200 U/mL in premenopausal and > 35 U/mL in post-menopausal patients), fixed or nodular pelvic mass, metastatic disease, ascites, or a strong family history of breast or ovarian cancer [5,6]. These guidelines appear to have a high sensitivity in addressing advanced stage ovarian cancer. However, the United Kingdom Collaborative Trial of Ovarian Cancer Screening (UKCTOCS) suggests that longitudinal algorithms are significantly superior compared to a simple CA 125 cutoff value. Serial changes in biomarkers for longitudinal algorithms are more sensitive in the early detection of invasive epithelial ovarian cancer. As the authors state, though, use of this screening algorithm is not indicated in low-risk women as there is no evidence of a mortality benefit [4]. There is a low sensitivity in referring early stage disease, particularly in premenopausal women [6]. Although ACOG/SGO Joint Opinion Guidelines are available, it remains a guideline and not a standard of care potentially due to the fact that there is no reliable screening or diagnostic test for ovarian cancer validated in the general population [4,5].

The importance of history and physical is emphasized throughout the medical community to infer differential diagnoses. With the reliance of laboratory testing and imaging, as well as increasing obesity levels limiting pelvic exams, there is a declining trend of accurately diagnosing patients solely based on physical exams [7]. Despite technological developments in analyzing adnexal masses, studies have shown that subjective assessments performed by experienced physicians have high sensitivities and specificities and are superior to other available classification systems or algorithms [5,8,9,10,11,12].

The purpose of this study is to demonstrate how well general obstetricians and gynecologists (OB/GYN) are able to appropriately refer patients to gynecologic oncologists for malignant ovarian masses based on clinical presentation (history and physical (H &P), available tumor markers, and imaging reports), in the absence of ROMA, OVA-1 testing.

## 2. Materials and Methods

This was a retrospective study examining patients who had a diagnosis of an ovarian or pelvic mass according to recorded ICD-10 codes. An institutional review board (IRB) exemption was approved from our institution on June 26, 2019, project number 19:16. Patients were included in the study if they met the following criteria: (1) women over 18 years old who presented with an ovarian or pelvic mass, (2) imaging studies (pelvic ultrasound, CT, and/ or MRI) were performed to describe the mass, (3) if available, tumor markers (CA125, CEA, CA 19-9) were collected, and (4) pathology reports with final diagnosis were available. Patients were strictly excluded from the study if (1) women were less than 18 years old and (2) no pathology reports were available or if no final diagnosis was given.

A query of our institution’s electronic medical record system was performed by the hospital IT Department using the above criteria for patient care dates ranging from 1/2/2017 to 1/31/2019. A total of 148 patients were identified as being eligible for inclusion in this study. Once the appropriate charts were identified, researchers recorded each of the patient’s history of presenting illness (with slight modifications to de-identify patients with minimal alteration to clinical information), listed available tumor markers, and copied available imaging reports, which combined listed details that were no longer than 1–1.5 pages per patient.

This information was then given to three board certified general OB/GYNs at our institution. Pathology reports were recorded separately and never given to the reviewers. Each of the three reviewers was then asked to determine whether or not they would refer the patient to a gynecologic oncologist. If yes, reviewers were asked to indicate the influencing factor(s) for referral (H&P, imaging report, tumor markers, or a combination). Lastly, reviewers denoted if they suspected the final diagnosis to be benign or malignant. An option of “not sure” was not listed for reviewers. The purpose of this study was to emulate a clinical scenario. In addition, researchers reasonably assumed that if reviewers were uncertain of a possible diagnosis, they would likely err on the side of caution and refer to avoid a misdiagnosis. Answers provided from the reviewers were then compared to the official diagnosis listed on the pathology report.

All statistical analyses were conducted using SAS 9.4 to determine the overall accuracy of the OB/GYN reviewers in referring suspected malignant cases to gynecologic oncologists based solely on the clinical presentation and provided imaging. The sensitivity, specificity, negative predicted value, and positive predictive value were calculated for each of the reviewers.

## 3. Results

Of the 148 patient records that were reviewed, 14 (9.5%) required referrals to a gynecologic oncologist for the malignant tumor that was present on the pathology report. As shown in Table 1, Reviewers #1, #2, and #3 all over-diagnosed the number of malignant cases, with Reviewers #2 and #3 both having a malignant diagnosis rate that was significantly higher than the actual rate of 9.5% (both *p* < 0.001). The same was true for the overall suggested referral rates, except that Reviewer #1 also significantly over-referred their patient cases (all *p* < 0.001). In our study, there were no borderline cases reported. When it came to their suggested referral rates among the true malignant cases, Reviewers #2 and #3 correctly referred 11 (78.6%) of the 14 women to an oncologist, whereas Reviewer #1 only referred nine (64.3%) of the women, causing their rate to be significantly lower than expected (*p* = 0.01).

Given these results, a sensitivity, specificity, negative predicted value, and positive predictive value was calculated for each of the three reviewers to better understand their accuracy in both patient diagnosis and suggested referrals, as shown in Table 2A,B.

In comparison to Reviewers #2 and #3, Reviewer #1 had the lowest sensitivity in both patient diagnosis (50.0%) and proper patient referrals (71.4%), indicating a lower probability that this reviewer would correctly identify that a patient’s tumor is malignant or needs referral compared to the other reviewers. However, in contrast, this reviewer had the highest specificity for both outcomes, indicating their higher probability to correctly identify benign cases (89.6%) and avoid over-referral of those benign cases (82.1%). All three reviewers had low positive predictive values (25.0–33.3%) and extremely high negative predictive values (94.5–98.0%) across both outcome measures. Thus, this implies a low probability of cases being malignant, or needing referral, when indicated by the reviewers’ response and a high probability that the cases were benign, or did not need referral, when reported by the reviewers. In terms of overall accuracy, Reviewers #2 and #3 were very similar in their ability to accurately predict patient diagnoses (81.1% vs 82.4%, respectively), while Reviewer #1 was slightly higher at 85.8%. The spread in accuracy was much larger for the suggested referrals where Reviewer #1 led with 81.1%, Reviewer #2 had a rating of 77.7%, and Reviewer #3 trailed with an overall accuracy of 74.3%.

When reviewing the determining factors for referral, Reviewer #1 indicated that a combination of H&P and imaging were the primary reasons for referral. Of those patients with malignant disease, 100% of referrals were due to a combination of H&P and imaging. Reviewer #2 referred 72.7% of patients with a malignant disease based on imaging with either a combination of H&P or tumor markers. Reviewer #3′s indication for referring patients with a malignant disease was based primarily on imaging (81.8%). It is important to note that CA 125 was not routinely ordered and therefore only 9 of 14 malignant cases had a CA 125 value. Of those nine cases, four of these patients had a normal CA 125. When examining the imaging modalities, 62 had pelvic ultrasounds alone, 14 had CT alone, 10 had MRI, and 62 had a combination of two imaging modalities, one of which included a pelvic ultrasound.

## 4. Discussion

An important prognostic factor for long-term survival in patients diagnosed with an ovarian mass is to be managed by a gynecologic oncologist, optimizing both surgical and medical management [13]. In our study, OB/GYNs refer between 23–32% of ovarian masses to a gynecologic oncologist with only 9.5% of cases found to be malignant. These high referral rates may potentially be due to differences in experience and comfort levels when operating on patients with an ovarian mass. Despite a high rate of referrals, OB/GYNs have a 74–81% sensitivity in accurately referring patients with a malignant disease. A high rate of referrals is not detrimental as more women with ovarian malignancies may be evaluated by gynecologic oncologists, while a false-positive diagnosis will not affect survival. In fact, Gostout and Brewer state that although no nationwide survey has been published, studies have shown that only 42% to 48% of women with ovarian malignancies are referred to a gynecologic oncologist. An even lower rate of referrals is made for women located in rural versus urban areas.

In this study, Reviewer #1 is primarily a hospitalist with an emphasis in obstetrics with 17 years of post-training clinical experience. Their accuracy in diagnosing benign cases was much higher compared to Reviewers #2 and #3 with an appropriately low referral rate as the majority of cases reviewed were benign. Reviewers #2 and #3 both specialize primarily in gynecology with minimal exposure to obstetrics. Reviewer #2 is a minimally invasive gynecologic surgeon with a special emphasis in the treatment of endometriosis and infertility with 16 years of experience. Reviewer #3 has been a gynecologic surgeon in practice for about 30 years and has trained and worked with gynecologic oncologists. Given this contrast, it is also appropriate that Reviewers #2 and #3 had similar referral rates for malignant cases and were comparatively more accurate in diagnosing malignant cases. Clinical experience is a key difference among the reviewers that can significantly skew results [2,5,9,10,11]. This study suggests that experienced OB/GYNs with an emphasis in gynecology surgery are more accurate in diagnosing and appropriately referring malignant cases to a gynecologic oncologist.

Generalists were about 81–85% accurate in diagnosing patients with benign or malignant cases at this institution. This distinction is important as it implies that OB/GYNs are able to appropriately refer patients with malignant masses to gynecologic oncologists using clinical presentation and imaging. A combination of H&P in congruence with imaging were the main indications used by the generalists for referrals, with imaging being a predominant factor notably for Reviewer #3. In fact, tumor markers were not a large factor for referral. CA 125 results were limited and CA125, ROMA, and OVA-1 were not routinely ordered for the patients selected. Studies have demonstrated that a generalist’s expertise in subjective assessment with ultrasound findings may offer a better “diagnostic certainty” in differentiating between benign and malignant masses compared to tumor markers and algorithms [2,11,12,13]. Although tumor markers are widely used, many researchers have also proved that women with ovarian cancer can have a normal CA 125 level [2,5]. Our data showed that of the malignant cases, 44.4% had a CA 125 that was normal. Our study suggests experienced clinicians can rely on sound clinical judgment and imaging to appropriately refer malignant ovarian masses in the absence of ROMA and OVA-1. It is important to note that our center is not a cancer institution. Therefore, decisions made by our generalists may potentially emulate real world decisions made on a daily basis by the vast majority of OB/GYNs in the United States.

Despite new advances, there is currently no acceptable screening tool for ovarian cancer or a standardized protocol for referral to gynecologic oncologists. The International Ovarian Tumor Analysis Group (IOTA) proposed “Simple Rules (SR),” an objective classification system used for triaging ovarian masses. A total of ten ultrasonographic features are classified as malignant, benign, or indeterminate characteristics [14]. SR does not rely on tumor markers and has fewer surgical interventions with no decrease in diagnosing ovarian malignancies compared to the Risk of Malignancy Index (RMI) [14]. SR is considered the best tool used to assess adnexal masses prior to surgery [11]. SR has been widely validated in Europe but has received limited acceptance in the United States and Canada [14]. Therefore, a newly published article establishes a design for risk stratification of ovarian masses incorporating a pattern-based approach used in both North America and Europe called the Ovarian-Adnexal Reporting and Data System (O-RADS). O-RADS scoring system ranges from 1 to 5 with varying percentages of the risk that an ovarian mass is malignant. Tumor markers also do not play a role in the evaluation; however, this should be individualized per patient. O-RADS is the only system that provides clear indications to refer to gynecologic oncologists and incudes risk categories as well as associated management [15]. However, O-RADS score is not yet standard on imaging reports in the United States.

Limitations of this study include potential bias. Although all patient identifiers were removed, some patients included in the study received care from one of the reviewers. Selective recall or familiarity of the cases may have potentially interfered with responses per clinical case. In addition, tumor markers were limited and not readily available. This is a potential area of discrepancy as to why tumor markers were not largely influential for referrals to a gynecologic oncologist. A suggested follow up study would compare the predicative value of detecting malignancy based on history and physical, imaging, and ROMA or OVA-1 factors. Another potential future study would be to have reviewers specifically indicate which factors from different protocols for referral to a gynecologic oncologist (ACOG/SGO Joint Opinion Guideline versus SR versus O-RADS) were used for the referral of adnexal masses. Thus, a comparison could be made to determine which guideline can improve the accuracy and sensitivity of appropriate referrals to a gynecologic oncologist. In doing so, a standard protocol for adnexal referrals may be established. A large-scale study with an increase in the number of reviewers is indicated to further validate conclusions drawn at this institution.

## 5. Conclusions

OB/GYNs in this study showed a tendency to over-refer patients to a gynecologic oncologist with adnexal masses. However, given the desire not to misdiagnose a deadly disease such as ovarian cancer, over-referral may be appropriate. Despite the high referral rates, generalists have a high degree of sensitivity in accurately referring malignant diseases based solely on clinical experience and imaging studies, which can improve survival rates with early intervention by gynecologic oncologists.

## Figures and Tables

**Table 1:** Reviewer diagnosis and referral results compared to the gold standard pathology reports.

Measure	Pathology Results	Reviewer 1	Reviewer 2	Reviewer 3
Benign Diagnosis	134 (90.5%)	127 (85.8%)	112 (75.7%) *	114 (77.0%) *
Malignant Diagnosis	14 (9.5%)	21 (14.2%)	36 (24.3%) *	34 (23.0%) *
Referrals Suggested	14 (9.5%)	34 (23.0%) *	41 (27.7%) *	48 (32.4%) *
Referrals Suggested Among True Malignant Cases	14 (100.0%)	9 (64.3%) *	11 (78.6%)	11 (78.6%)

Significant *p*-values (< 0.05) are indicated with (*).

**Table 2:** (**A**) Reviewer accuracy in patient diagnosis. (**B**) Reviewer accuracy in suggested patient referrals.

**A**			
**Accuracy Measure**	**Reviewer 1**	**Reviewer 2**	**Reviewer 3**
Sensitivity (95% CI)	50.00% (23.0%, 77.0%)	78.60% (49.2%, 95.3%)	78.60% (49.2%, 95.3%)
Specificity (95% CI)	89.60% (83.1%, 94.2%)	81.30% (73.7%, 87.6%)	82.80% (75.4%, 88.8%)
PPV (95% CI)	33.30% (19.6%, 50.7%)	30.60% (22.0%, 40.8%)	32.40% (23.2%, 43.2%)
NPV (95% CI)	94.50% (91.1%, 96.7%)	97.30% (93.0%, 99.0%)	97.40% (93.1%, 99.0%)
Overall Accuracy (95% CI)	85.80% (79.1%, 91.0%)	81.10% (73.8%, 87.1%)	82.40% (75.3%, 88.2%)
**B**			
**Accuracy Measure**	**Reviewer 1**	**Reviewer 2**	**Reviewer 3**
Sensitivity (95% CI)	71.40% (41.9%, 91.6%)	78.60% (49.2%, 95.3%)	85.70% (57.2%, 98.2%)
Specificity (95% CI)	82.10% (74.5%, 88.2%)	77.60% (69.6%, 84.4%)	73.10% (64.8%, 80.4%)
PPV (95% CI)	29.40% (20.3%, 40.5%)	26.80% (19.5%, 35.8%)	25.00% (19.0%, 32.2%)
NPV (95% CI)	96.50% (92.3%, 98.4%)	97.20% (92.7%, 99.0%)	98.00% (93.1%, 99.4%)
Overall Accuracy (95% CI)	81.10% (73.8%, 87.1%)	77.70% (70.1%, 84.1%)	74.30% (66.5%, 81.2%)
